# Cancer Niches and Their Kikuchi Free Energy

**DOI:** 10.3390/e23050609

**Published:** 2021-05-14

**Authors:** Noor Sajid, Laura Convertino, Karl Friston

**Affiliations:** 1 Wellcome Centre for Human Neuroimaging, Institute of Neurology, University College London, London WC1N 3BG, UK; k.friston@ucl.ac.uk; 2 Institute of Cognitive Neuroscience, University College London, London WC1N 3BG, UK

**Keywords:** cancer niches, free energy, Kikuchi approximations, apoptosis, metastasis, cluster variation method

## Abstract

Biological forms depend on a progressive specialization of pluripotent stem cells. The differentiation of these cells in their spatial and functional environment defines the organism itself; however, cellular mutations may disrupt the mutual balance between a cell and its niche, where cell proliferation and specialization are released from their autopoietic homeostasis. This induces the construction of cancer niches and maintains their survival. In this paper, we characterise cancer niche construction as a direct consequence of interactions between clusters of cancer and healthy cells. Explicitly, we evaluate these higher-order interactions between niches of cancer and healthy cells using Kikuchi approximations to the free energy. Kikuchi’s free energy is measured in terms of changes to the sum of energies of baseline clusters of cells (or nodes) minus the energies of overcounted cluster intersections (and interactions of interactions, etc.). We posit that these changes in energy node clusters correspond to a long-term reduction in the complexity of the system conducive to cancer niche survival. We validate this formulation through numerical simulations of apoptosis, local cancer growth, and metastasis, and highlight its implications for a computational understanding of the etiopathology of cancer.

## 1. Introduction

The human body develops via the progressive specialisation of pluripotent stem cells, niche construction, and organ development (i.e., morphogenesis). Understanding these processes is not only fundamental to understanding how multicellular organisms come to be, but also has important implications for aberrant events. Cellular de-differentiation can cause (and be a consequence of) disruption of the mutual balance between cells and their niche, where cell proliferation and genetic regulation are released from their autopoietic homeostasis. The disruption of this balance and the creation of new niches drives cancer cell growth and sustains their survival. The concept of a stem cell niche [1,2] identifies the “whole” of the wholeness of the microenvironment in which stem cells differentiate, grow, and survive, and the humoral, paracrine, physical, metabolic, neuronal, structural properties with which the cell exchanges information. These specialised microenvironments are found in adult organisms and their homeostatic disruption may facilitate the development of cancer colonies through an interaction between cells and their niches [3]. In this paper, we pursue the notion that cancer niche construction requires individual cells with oncogenic potential to interact in subpopulations (i.e., clusters) of healthy and cancerous cells to reach a particular attractor state.

Briefly, cancer cell niche construction results from a maladaptive degeneration of the complex dynamic homeostasis that undergirds the balanced survival, renewal, and repair of mature organs. This homeostasis is replaced by a new niche that is beneficial to cancer growth, without which oncogenic cells might not be able to escape the physiological mechanisms of control and elimination of the degenerated cells [4]. We acknowledge the phenotypical differences between cancer cells and cancer stem cells, but for simplicity, we operationalise all cell types as simply cancer cells. Note, that cancer stem cells are a small subpopulation of cancer cells with capability of self-renewal, differentiation, and tumorigenesis [4,5].

To become carcinogenic, cells acquire some key properties that free them from their physiological life cycle. The main properties are: (i) self-sufficiency in growth signals; (ii) insensitivity to anti-growth signals; (iii) tissue invasions and metastasis; (iv) limitless reproductive potential; (v) angiogenesis; and (vi) avoiding apoptosis [6]. It may not be necessary for a cell to acquire all these properties to become a cancer cell, since different combinations of these properties are sufficient for inducing oncogenesis. For example, only some cancers have metastatic properties, namely the ability to leave the tissue of origin, move to another location and colonise another organ. The presence of metastatic sites determines the progression of cancer, staging, and patient prognosis. Fortunately, healthy organisms can fight oncogenesis via different mechanisms of self-preservation. Among these, apoptosis (i.e., programmed death) is crucial. This is a controlled mechanism of cell death that intervenes in normal organ repair, growth, renewal, as well as in inflammation and cancer.

For a computational understanding of cancer niche construction, a 2D Ising model has been used to evaluate phase transitions between cancerous and healthy cells [7,8,9]. For example, [10] used the Ising Hamiltonian to model metastasis, where the updates in the energy function were modelled using mean-field approximations, i.e., only considering average interactions. This factorised approach is consistent with other applications of the Ising model for simulating cancer progression [7,8,9]; however, cell niches are the result of numerous mechanisms, both at the cellular and population level. The single cells are involved in the construction and maintenance of the niche and they adapt via genetic, epigenetic, metabolic, structural, internal, and external signalling mechanisms. More recently, theoretical work has underlined the fundamental role of population dynamic and mutual information exchange in guiding the fate of local subpopulations of cells and the niche as a whole [11,12,13] via cell-to-cell, cell-to-niche, and niche-to-cell information flow.

Consequently, our work builds upon previous computational approaches by (i) casting cancer niche construction as a direct consequence of interactions between clusters of cancer and healthy cells, and (ii) using a Kikuchi Hamiltonian as a way to account for higher-order interactions when evaluating the state functions of such systems [14,15,16,17,18,19]. This allows us to move away from thinking about cells in isolation and towards accounting for interactions within cancer niches. Briefly, Kikuchi formulation approximates the free energy as a sum of local free energies of a cluster of cells (or nodes) in the system. In doing so, it provides a way to define a local population (or base cluster) which includes all the interactions between the cells.

In what follows, we simulate three cancer trajectories to provide proof of the principle of a computational formulation for oncogenic etiopathology. For this, we simulate a 2D Ising model for evaluating how cancer trajectories unfold using Kikuchi free energy approximation. Explicitly, we manipulate four (hyper)parameters, namely an interaction parameter that regulates the type of cell interaction, a tolerance parameter that determines the acceptance level of cancerous cells, a growth parameter that regulates the number of cell states switched during a single trial, and a noise parameter that influences the transition dynamics. We describe the technical details in Section 3.

First, we simulate local cancer growth within the tissue of origin by allowing pairwise interactions between cancerous and healthy cells (i.e., a high interaction parameter value). This allows us to reproduce the homeostatic shift in a healthy organ that results from the acquired oncogenic properties of single cells within it, but it is crucially sustained and enabled by the broader dynamic at the sub-population level. Secondly, we model the metastasis where cells exit their primary site and invade secondary locations. Here, cells have to prepare for the new environment before arrival in the metastatic site. Without a favourable environment, the cells would not be able to colonise the new organ. We treat this cancer spread as an embedded mechanism in the niche construction process. This is achieved by allowing pairwise interactions between/across cancerous and healthy cells (i.e., low positive interaction parameter value), alongside a restricted capacity to grow outside the initial cancer site (i.e., decaying noise parameter). Finally, we consider apoptosis, namely the programmed death of the cancer cells. In our simulations, we assume that the cancer cells do not acquire the ability to escape apoptosis, and the physiological mechanisms of control activate to eliminate the pathological cells (via a low tolerance threshold).

The remainder of the paper is organised as follows. In Section 2, we briefly review the notion of free energies, specifically their relevance in evaluating systems under thermodynamic equilibrium. We then describe and provide simple examples for the use of Kikuchi approximations in evaluating a system state function. Equipped with this, Section 3 describes the system used to simulate cancer niche construction, and the parameters required to simulate cancer progression. Section 4 details the simulation results for the three cancer trajectories. We conclude by highlighting the implications for specific etiopathology, and potential future directions.

## 2. Free Energies

In this section, we review the notion of free energy and its relevance for modelling cancer niche construction. For this, we follow the formulation introduced in [20]. Briefly, free energy is the state function of a (random dynamical) system that possesses a steady-state or pullback attractor. Its value is determined by the current state of the system. The free energy can be used to describe the spatiotemporal evolution of a system as it converges to an equilibrium or nonequilibrium steady state, e.g., a particular tissue population in the human body or the body itself. The distinction between nonequilibrium and equilibrium steady state rests upon the presence of solenoidal or divergence free flow. In the absence of solenoidal dynamics, the flow of systemic states is entirely dissipative, and the steady state is at (thermodynamic) equilibrium.

**Figure 1:**
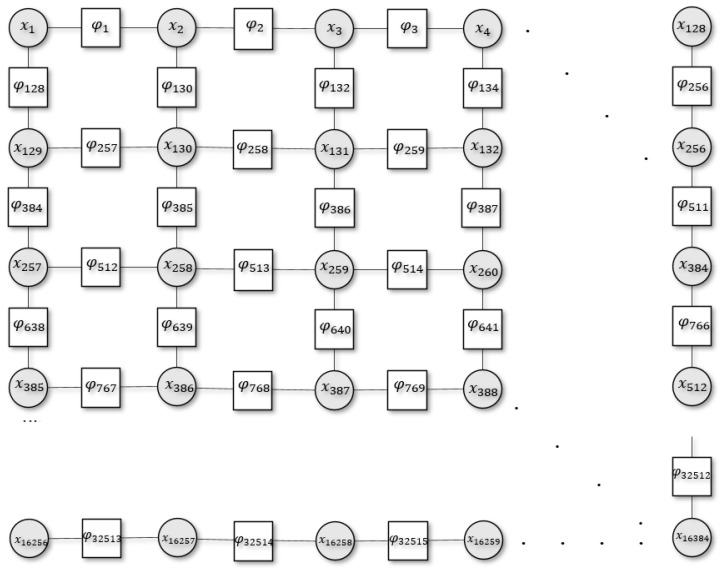
Schematic illustration of the 2D system with a set of N discrete random variables where $N=128^{2}$. Here, the circles denote the variable node for each variable ($x_{i}$), squares denote the factor nodes ($\varphi_{a}$), and edges connect the variable node i to the factor a.

To make this concrete, we introduce a closed 2D system (e.g., an Ising model) with a set of N discrete random variables, $\left\{X_{1},X_{2},X_{3},\ldots ,X_{N}\right\}$ arranged in a square grid graph (Figure 1). Each random variable, $X_{i}$, has a possible realisation $x_{i}$. Here, we cast these realisations as distinct cell states in the body, or a particular tissue population, that can be either healthy or cancerous. The overall system state is denoted by the vector $x=\{x_{1},x_{2},\ldots ,x_{N}\}$, with the corresponding energy of the system given by its Hamiltonian, H(x). For computational ease, we deal with an effective Hamiltonian to represent the system in a reduced space through nonlinear averaging of the true Hamiltonian. Consequently, this only describes a part of the eigenvalue spectrum of the true Hamiltonian. This formulation is in contrast to the molecular Hamiltonian where the Hamiltonian is decomposed into two or more separable parts (i.e., nuclei and electrons), and their interactions. For our purposes, this separation could involve the inclusion of additional random variables (that represent external states or distinct internal states) (z) with a Hamiltonian of the following form: $H=H_{1}(x)+H_{2}(z)$. Practically, decompositions of the Hamiltonian of this sort are potentially important because they could naturally account for local interactions; however, their inclusion would not speak to the long-range interactions that are the current focus.

At steady state, the probability of finding the system in this state is given by Boltzmann’s law, which can be expressed as [21]:

(1) $$\begin{array}{cc} P(x) & =\frac{1}{Z}e^{-\beta H(x)}\\ \ln P(x) & =-\beta H(x)-\ln Z\\ Z & ≜\sum_{x\in M}e^{-\beta H(x)} \end{array}$$

where β is the inverse temperature or precision that shapes the probability density of the distribution, Z is the partition function, and M is the set of all possible states of the system. For our purpose, we make simplifying assumptions about our system. and set β=1. Briefly, we assume that the joint probability distribution describes some nonphysical system. This allows us to view Boltzmann’s law as a way of defining the energy of the system where is simply an arbitrary unit that scales the energy measure [20].

The partition function, Ζ, is closely related to Helmholtz free energy, $F_{H}$ [20,22,23]:

(2) $$\begin{array}{cc} F_{H} & =-\ln Z\\ & =\ln P(x)+H(x) \end{array}$$

This quantity can be approximated using variational calculus [24], which allows an otherwise intractable $F_{H}$ to be approximated. This requires the introduction of a variational distribution, Q(x), assuming $\sum_{x\in M}Q(x)=1$ and 0≤Q(x)≤1;∀x, and the corresponding variational (or Gibbs) free energy, $F_{V}$ [20]:

(3) $$\begin{array}{cc} F_{V} & \equiv \underset{Internal\;energy}{\underset{︸}{U(Q)}}-\underset{Entropy}{\underset{︸}{S(Q)}}\\ & =\sum_{x\in M}Q(x)H(x)-\left[-\sum_{x\in M}Q(x)\ln Q(x)\right]\\ & =\sum_{x\in M}Q(x)[\ln Q(x)-\ln P(x)]+F_{H}\\ & =\underset{Complexity}{\underset{︸}{D_{KL}\left[Q(x)||P(x)\right]}}+F_{H} \end{array}$$

where $H(x)=-\ln P(x)+F_{H}$, and $D_{KL}$ is the Kullback–Leibler divergence. We know that $D_{KL}\left[Q(x)||P(x)\right]\geq 0$ [25], hence $F_{V}$ is an upper bound on the $F_{H}$:

(4) $$F_{V}\geq F_{H}$$

Thus, it follows that by minimising $F_{V}$ with respect to Q(x), we can get a good approximation of $F_{H}$ and recover p(x); however, as N→c,c≫1, this also becomes intractable. Therefore, a practical solution is to consider the upper bound $F_{H}$ by minimising $F_{V}$ with respect to a restricted class of variational distributions Q(x). A standard restriction over the variational distribution follows from the mean-field approach, i.e., an absence of interactions [26,27,28]:

(5) $$\begin{array}{cc} Q_{MF}(x) & =\prod_{i=1}^{N}Q_{i}(x_{i})\Rightarrow \\ F_{MF} & =U_{MF}(Q)-S_{MF}(Q)\\ & =-\sum_{a}^{}\sum_{x_{a}}\ln\varphi_{a}(x_{a})\prod_{i\in N(a)}Q_{i}(x_{i})+\sum_{i=1}^{N}\sum_{x_{i}}Q_{i}(x_{i})\ln Q_{i}(x_{i})\\ H(x) & =-\sum_{a}^{}\ln\varphi_{a}(x_{a}) \end{array}$$

where the Hamiltonian is defined as the sum of its factors, $\varphi_{a}$, under a factor graph probability distribution function (Figure 1). Conversely, we could consider more complicated factorisations, like structure mean-field approaches [29], Bethe free energy [15,30], or Kikuchi free energy approximations or a cluster variational method [16,17], which can provide more accurate approximations. Interestingly, the Kikuchi free energy is a generalisation of the Bethe free energy, using higher-order approximations [17].

### Kikuchi Free Energy

In this work, we use Kikuchi approximation to evaluate the free energy of the system [15,16,17,20,31]. This allows us to account for higher-order interactions between variable nodes, mimicking the type of interactions present during cell niche construction. Practically, this characterises the system evolution in terms of interactions between neighbours of cancerous and health nodes, up to size d. This is calculated as changes to the sum of the energies of baseline clusters of variable nodes, minus the overcounted interactions (and interactions of interactions). The premise is that in accounting for higher-order interactions within clusters, we can get a better evaluation of the systems overall state function (i.e., free energy).

Formally, the Kikuchi free energy $F_{K}$ is:

(6) $$F_{K}=\sum_{r\in R}c_{r}F_{r}$$

where:

(7) $$\begin{array}{cc} F_{r} & =U_{r}(Q)-S_{r}(Q)\\ & =\sum_{x_{r}}Q_{r}(x_{r})H_{r}(x_{r})+\sum_{x_{r}}Q_{r}(x_{r})\ln Q_{r}(x_{r})\\ H_{r}(x_{r}) & =-\sum_{a\in A_{r}}\ln\varphi_{a}(x_{a}).\\ c_{r} & =1-\sum_{s\in super(r)}c_{s} \end{array}$$

where r denotes a region, i.e., the set of variable nodes within a cluster of size d, and R a finite set of all possible regions. Additionally, $c_{r}$ is the overcounting number of regions, and super(r) is the set of all super-regions of r. This follows from how $F_{K}$ is approximated. Generally, for each factor node within a cluster we need to include all adjacent variables nodes and sum the computed free energy; however, this process results in repeated counting of the interactions between sets within a cluster. Therefore, we need to subtract the free energy of interactions and interactions of interactions.

**Figure 2:**
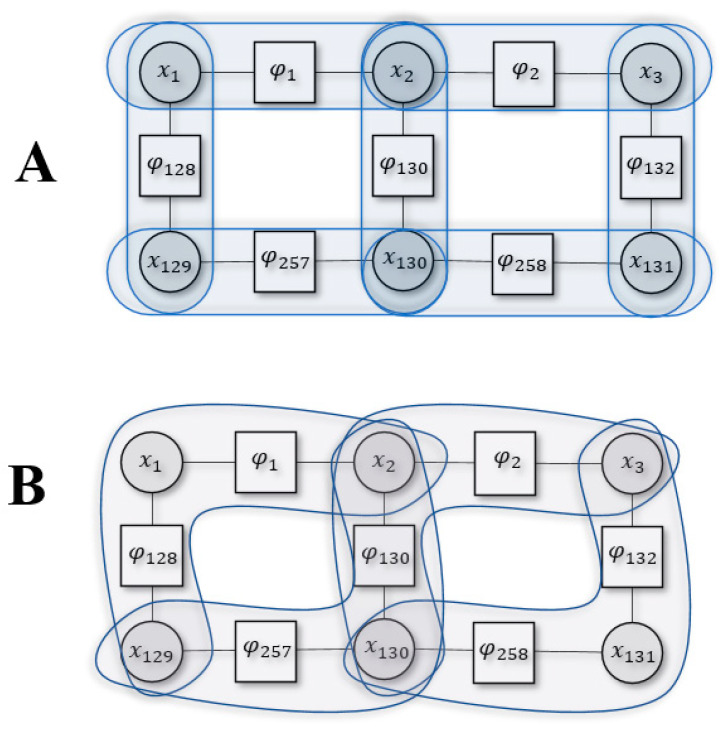
Example of Kikuchi free energy with two different cluster sizes. Panel **A** is for d=2 or Bethe approximation and panel **B** is for d=3.

Figure 2 shows an example Kikuchi approximation using part of the system defined in Figure 1 for two different cluster sizes (2,3). To evaluate the Bethe approximation (i.e., d=2) we use pairwise clusters. The Bethe entropy would then be the sum of all entropies in the pairwise cluster, subtracting the overcounted cluster interactions [15]:

(8) $$\begin{array}{cc} S_{d=2}(x) & =S(x_{1,2})+S(x_{2,3})+S(x_{129,130})+S(x_{130,131})\\ & +S(x_{1,129})+S(x_{2,130})+S(x_{3,131})\\ & -S(x_{1})-S(x_{129})-S(x_{3})-S(x_{131})\\ & -2S(x_{2})-2S(x_{130})\\ S & =-\sum_{x_{i}}Q(x_{i})\ln Q(x_{i}) \end{array}$$

where $x_{1}$ appears in two clusters, $\left\{x_{1,2}\},\{x_{1,129}\right\}$, i.e., its entropy was overcounted only once and we subtract it once. Conversely, $x_{2}$ appears in three clusters, $\left\{x_{1,2}\},\{x_{2,3}\},\{x_{2,130}\right\}$, and this mean that its entropy was overcounted twice, and we subtract it twice.

We could approximate $F_{K}$ differently by using a cluster size of 3:

(9) $$\begin{array}{cc} S_{d=3}(x) & =S(x_{129,1,2})+S(x_{129,130,2})+S(x_{130,2,3})+S(x_{130,131,3})\\ & -S(x_{2,129})-S(x_{3,130})-S(x_{2,130}) \end{array}$$

Here, $x_{129}$ and $x_{2}$ appear together in two separate clusters $\left\{x_{129,1,2}\},\{x_{130,129,2}\right\}$, so their entropy is subtracted. Similar logic follows for $\left(x_{130},x_{3}\right)$ and $\left(x_{130},x_{2}\right)$.

We can define the Kikuchi Hamiltonian based on similar intuition. Accordingly, the Hamiltonian for Figure 2B, found using Equation (7), is the following:

(10) $$\begin{array}{cc} H_{d=3}(x) & =H(x_{129,1,2})+H(x_{129,130,2})+H(x_{130,2,3})+H(x_{130,131,3})\\ & -H(x_{2,129})-H(x_{3,130})-H(x_{2,130}) \end{array}$$

Generally, the approximation accuracy is improved when we consider larger cluster sizes, which is in contrast to mean-field formulation. Explicitly, for d=N, the Kikuchi approximation for the entropy becomes exact [32]; however, by working with clusters, we cannot define an overall variational distribution, Q(x), that is consistent with $Q_{r}(x_{r})$ and are unable to obtain an upper bound for $F_{H}$ [33].

## 3. Constructing Cancer Niches using Kikuchi Free Energy

In this section, we introduce the model used for simulating cancer niches using Kikuchi free energy approximation. For this, we work with the system briefly introduced in Section 2 (Figure 1). Explicitly, this is a 2D Ising model with $N=128^{2}$ discrete variables, $\left\{X_{1},X_{2},X_{3}\ldots ,X_{N}\right\}$, and is arranged in a grid structure. These variables, $X_{i}$, represent individual cells in a particular tissue population, and each variable can be in one of two states $x_{i}\in \{0,1\}$. Here, 0 denotes a healthy state, i.e., $x_{i}^{o}$, and 1 denotes a cancerous state i.e., $x_{i}^{1}$. We factorise the 2D system as follows:

(11) $$P(x)=\sum_{s\in \{0,1\}}\left(\sum_{i,j\in N}P(x_{{}_{i,j}}^{s},x_{{}_{i,j+1}}^{s},x_{{}_{i-1,j}}^{s},x_{{}_{i-1,j+i}}^{s})\right)$$

where i,j denote the position on the grid (row, column) and s represents the particular variable realisation. This factorisation is a simplified characterisation of sub-populations of cells interacting during cancer niche construction. It is a simple characterisation because it corresponds to interactions of size 4. A different factorization of the system could have been chosen that might require a different higher-order Kikuchi approximation. Technically, even this simple construction leads to non-unique factorisation because of boundary effects. Consequently, to approximate the free energy of this factorised system we need to account for higher-order interactions whilst accounting for overlapping interactions. Specifically, we specify a Kikuchi Hamiltonian to evaluate the energy exchange in these interacting nodes. Practically, this is achieved by using the Kikuchi formulation introduced in for d=3 [14,16] or $B_{3}$ using [16]’s notation. This is appropriate because $B_{3}$ with a base cluster as an angle of size 3 gives the same results when using a base cluster of size 4 but with a square [16]:

(12) $$\begin{array}{cc} F_{V} & =U_{K}(Q)-S_{K}(Q)+\lambda_{1}\left(1+\sum_{s_{1},s_{2},s_{3}\in \{0,1\}}\sum_{i,j}H(x_{{}_{i,j}}^{s_{1}},x_{{}_{i-1,j-1}}^{s_{2}},x_{{}_{i-1,j+i}}^{s_{3}})\right)\\ & +4\lambda_{2}\left(\begin{array}{l} Q(x_{{}_{i,j}}^{0},x_{{}_{i-1,j-1}}^{1},x_{{}_{i-1,j+i}}^{0})+Q(x_{{}_{i,j}}^{1},x_{{}_{i-1,j-1}}^{1},x_{{}_{i-1,j+i}}^{0})+Q(x_{{}_{i,j}}^{0},x_{{}_{i-1,j-1}}^{1},x_{{}_{i-1,j+i}}^{1})\\ -Q(x_{{}_{i,j}}^{0},x_{{}_{i-1,j-1}}^{0},x_{{}_{i-1,j+i}}^{1})-Q(x_{{}_{i,j}}^{1},x_{{}_{i-1,j-1}}^{0},x_{{}_{i-1,j+i}}^{0})-Q(x_{{}_{i,j}}^{1},x_{{}_{i-1,j-1}}^{0},x_{{}_{i-1,j+i}}^{1}) \end{array}\right) \end{array}$$

(13) $$U(Q)=\underset{=0}{\underset{︸}{U_{0}}}+\underset{Interaction}{\underset{︸}{\varepsilon_{IN}\left(Q\right)}}$$

See Kikuchi and Brush (1976) Table II and IV for graphical representations. Additionally, the Appendix A presents the exact Kikuchi free energy approximation (Equations (A1)–(A3)). Here, $\lambda_{1},\lambda_{2}$ are the Lagrangian multipliers necessary to satisfy the normalisation condition:

(14) $$1=\sum_{s_{1}\in \{0,1\}}Q(x_{}^{s_{1}})=\sum_{s_{1},s_{2},s_{3}\in \{0,1\}}\sum_{i,j}Q(x_{{}_{i,j}}^{s_{1}},x_{{}_{i-1,j-1}}^{s_{2}},x_{{}_{i-1,j+i}}^{s_{3}})$$

$\varepsilon_{IN}$ is the interaction parameter, and $U_{0}$ is the activation energy. Following [14,16], we set the activation energy to 0 and quantify the interaction energy using pairwise interactions (Equation (A2)). Consequently, this particular interaction parameter allows us to accommodate the type of interactions the system evinces. Intuitively, increasing the interaction energy encourages interactions across healthy and cancerous pairs, whilst lower interaction energy encourages interactions within healthy or cancerous pairs [14,18]. Perturbing this interaction energy has a direct impact on the overall free energy of the system. In doing so, we have a way to evaluate the free energy of the system for a given trial. Now, we formalise a setting for how particular realisations evolve (i.e., transition from one state to another) under this system. At each trial, J variables may update their current state from either cancer to healthy or healthy to cancer state. This determines the growth rate hyperparameter i.e., α=[JN]×100. The variables transition to the other state (i.e., either healthy or cancerous, given the previous state) determined by the probability ρ:

(15) $$\begin{array}{l} v\sim p(v_{t})=\rho^{v_{t}}{\left(1-\rho \right)}^{v_{t}}\\ x_{j,t}\sim \{\begin{array}{cc} x_{j,t}=x_{\tilde{j}\backslash j\in R,t}^{s_{t}} & \;\mathrm{if}\;v_{t}>\mathrm{nt},\\ x_{j\in N,t}^{s_{t}\neq s_{t-1}} & \;\mathrm{if}\;v_{t}\leq \mathrm{nt}, \end{array} \end{array}$$

where $v_{t}$ denotes an auxiliary Bernoulli random variable indicating whether the variable $x_{j}$ is in the same cluster, R, as $x_{\tilde{j}}$ at trial t, and nt is the noise hyperparameter. The variables $\tilde{j}$ are identified based on the tolerance, tol, threshold:

(16) $$x_{\tilde{j}}\sim \{\begin{array}{c} 1\;\mathrm{if}\;tol>0.5\\ 0\;\mathrm{if}\;tol\leq 0.5 \end{array}$$

**Figure A1:**
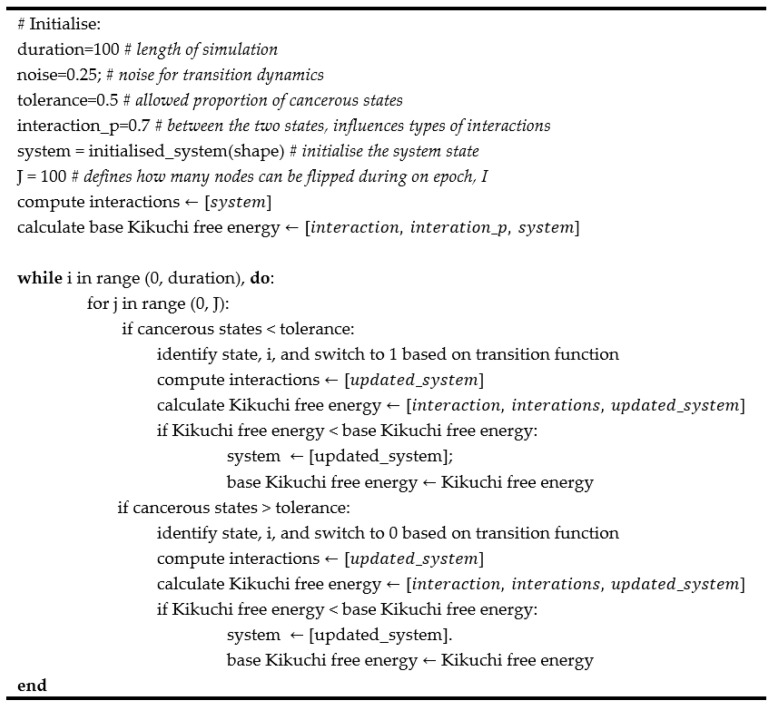
Pseudocode.

The tolerance hyperparameter controls the maximum proportion of cancerous states allowed in the system. In our deterministic formulation, these state updates are retained if they minimise the overall Kikuchi free energy of the system. See the Appendix B for the pseudocode (Figure A1).

### 3.1. Simulations

Using the above formulation, we simulated three cancer trajectories: local growth, metastasis, and apoptosis. The initialisation of the system is dependent on the simulation, and the specific parameterisations for each simulation are presented in Table 1.

#### 3.1.1. Local Growth

First, we modelled growth within the tissue of origin. This speaks to a modification of healthy cells within the original site, i.e., the healthy nodes now become cancerous. Simulating localised cancer cell growth is a core step to elucidating how cancer growth in a healthy organ can be the result of an energy-efficient process in terms of population dynamics although being pathological for the organism as a whole. To simulate this, we specified a high tolerance for cancerous nodes, along with a high energy interaction parameter (Table 1) that constrains the specification of $x_{j}$ to variables in the existing cancerous cluster. Here, the positive interaction parameter induces a reduction in overall free energy when cancerous cells interact with other cancerous cells.

For this cancer niche, we initialised the system with a single cancerous cell. Using this, we simulated two distinct patterns: dispersive and localised. The dispersive simulation emulates instances where growth is spread out throughout the tissue population. As a result, the noise hyperparameter was set to 0.25. Conversely, the localised simulation emulates instances where growth is restricted to a small region of the tissue population. For this, we set the noise hyperparameter to 0.

#### 3.1.2. Metastasis

We next modelled metastasis. Understanding metastatic spread is a fundamental priority in cancer research and treatment. With this simulation, we attempted to recreate in silico metastatic progression where the metastatic invasion of a distant organ is enabled by the re-creation of a new niche, without which the cells (although able to migrate) would not survive. Generally, the development of a metastasis occurs in several steps which can develop in a different order over time, including the creation of a premetastatic niche (induced by a distant tumour), mesenchymal transition (EMT) of the original mass (which provides invasive properties), degradation of basement membranes and remodelling of the extracellular matrix (ECM), invasion of the surrounding tissue, angiogenesis, intravasation, arrest of the tumour cells in a capillary bed, extravasation, and the development of macrometastasis [34].

**Figure 3:**
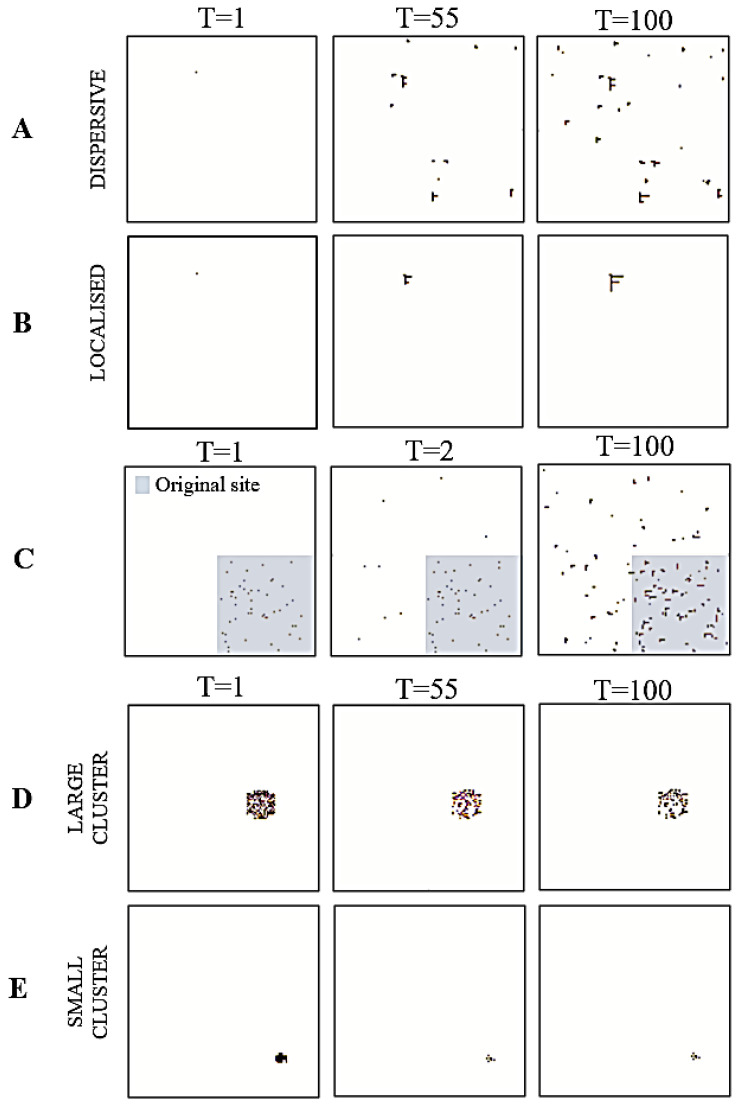
Simulating cancer niches. The figures are pictorial representations of the cancer niches in different trials during simulation. Each row denotes a different simulation: local growth (**A**,**B**), metastasis (**C**), and apoptosis (**D**,**E**). For row C, the grey square represents the original site location.

Intuitively, metastasis is the movement of cancer cells from a primary site, (e.g., the original skin tissue of growth) to a secondary location (e.g., lymph nodes). Here, we initialised a small region of the system as the primary site (Figure 3C, grey square), with the remaining grid representing the secondary location. We used a decaying noise hyperparameter to model the transition dynamics alongside an increasing growth rate. This allowed us to emulate the movement of cancerous cells from the primary site to the secondary location, via the bloodstream. Additionally, setting the interaction parameter value to 1.88 allowed us to strike a balance between having both (i) pairwise interactions across cancerous and healthy cells and (ii) pairwise interactions between cancerous and healthy cells within the cluster. This created a system with cancer cells in regions outside the primary site that could support the recreation of a new cancer niche.

#### 3.1.3. Apoptosis

We then modelled apoptosis. This is a controlled mechanism of cell death that intervenes in normal organ repair, growth, and renewal, as well as in inflammation and cancer. It can result from three pathways (extrinsic, intrinsic, and perforine/granzyme). In cancer proliferation, the intrinsic pathway is impaired and cancer cells can escape their death [35]. Here, we aimed to show how the organisms self-preservation mechanisms could be properly activated against the oncogenic cells. Accordingly, we did not expect cancer growth and expected the new niche to be unable to stabilise at a new homeostatic equilibrium.

During this process, the tissue population would normally go through several stages: (i) healthy cells becoming cancerous; (ii) cancerous cells dying; and (iii) remaining healthy cells multiplying and repairing the organ. From our perspective, this simply ensures switching off existing cancerous cells in the population and replacing them with healthy cells.

We were interested in evaluating how the size of the cancer niche impacted apoptosis. Accordingly, we initialised two distinct grids. One had a small cancerous cluster of 41 nodes (or 0.25% of the population), and the other had a larger cluster of 172 nodes (or 1% of the population). For both, we set the tolerance hyperparameter to 10^−5^ and the interaction parameter to −1.426 to simulate two instances of apoptosis. Here, having a negative interaction parameter induces a reduction in the overall Kikuchi free energy when more cancer cells interact with healthy cells.

Each parameterisation was simulated across 100 trials with ρ=0.2 and the other parameters were kept consistent.

## 4. Results

**Figure 4:**
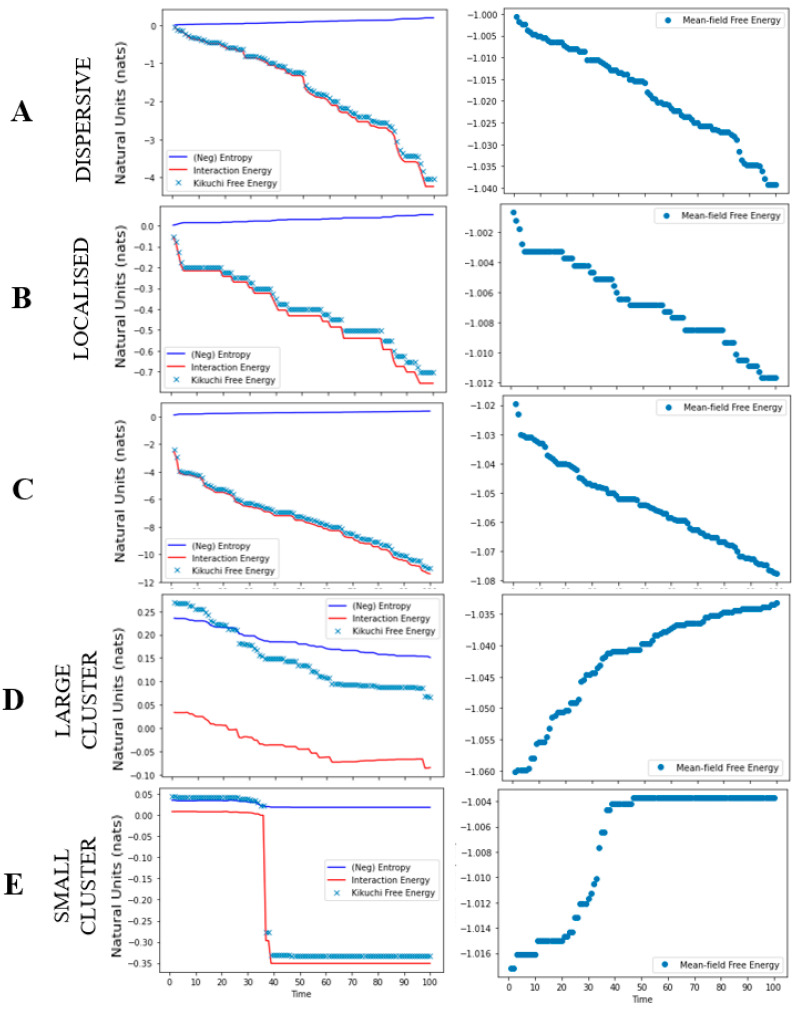
Free energy as a function of time. The graphic plots the free energies for each cancer niche simulation. Each row denotes a different simulation: local growth (**A**,**B**), metastasis (**C**), and apoptosis (**D**,**E**). For each plot, the Y-axis reports the free energy in natural units [36] and the x-axis as the trial. The first panel reports the Kikuchi free energy (blue dots), interaction energy (red line), and entropy (blue line). The second panel reports the mean-field free energy (blue dots).

The results from the simulations, for each cancer niche, are shown in Figure 3 and Figure 4. For the local growth simulation, the construction of the cancer niche was entirely consistent with our expectations. That is, from a single cancerous cell we could observe cancer development. Specifically, by setting a high interaction and tolerance parameter, the cancer was able to survive in healthy tissue. In other words, the overall free energy of the system was minimised by allowing for interactions across cancerous and healthy cells that lead to an oncogenic environment (i.e., the topology of our system) (Figure 4). The free energy results measured using Kikuchi and mean-field approximation presented similar path trajectories.

The metastasis simulation illustrates (i) the movement of cancerous cells away from the original site and (ii) the ability to sustain the ensuing changes in cell nodes outside the original nodes. (Figure 3). This is a direct result of the interaction parameter value, that allowed for across/between interactions of cancerous and healthy cells. Thus, any changes to the overall system, distal to the original site, were maintained because they minimised the overall Kikuchi free energy of the system. As in the local growth simulation, we see that both Kikuchi free energy and mean-field free energy approximations follow a similar trend (Figure 4); however, the Kikuchi free energy gradient was steeper over time.

For the apoptosis simulation, the system was unable to maintain the existing cancer niche and/or create a new cancer niche. This is reflected by the gradual decline in the overall number of cancerous cells. It is a direct result of reducing the tolerance threshold to 0.00001, where the system now updates the state of cancerous cells to healthy (i.e., a process of renewal and repair). Moreover, the negative interaction parameter value effectuates an overall minimisation of the Kikuchi free energy as the number of interactions between healthy and healthy cells increases. Ultimately, this leads to an apoptotic fate for cancer cells (Figure 4). Interestingly, although we observed a drop in the Kikuchi free energy (as expected), the mean-field free energy for this system increased over time.

## 5. Discussion

Organ development and cellular differentiation, de-differentiation, and niche homeostasis arise from a complex interaction between both deterministic and stochastic mechanisms [13]. In these processes, the spatial aspects are as important as the temporal ones, and steady system states are found when the free energy of spatiotemporal dynamics reach minima. In this context, the geometry of the cell’s population presents a role that goes beyond the mere effect on mechanics forces [37,38,39,40].

The original approach to investigate cellular population dynamics has mainly been deterministic, i.e., a stable genetic code defines cell fate as a programmed hierarchical process. More recently, research has shown how another group of mechanisms based on stochastic self-organisation plays a complementary role in morphogenesis (i.e., organ development) and cell development [41]. Under this perspective, genetic factors would interact with self-organisation mechanisms to balance the growth of an organism and the organisation of cellular assemblies in direct connection with environmental factors. The specific shapes and functions of cells are associated with physical constraints that, although not being genetically encoded, determine the spatial and functional organisation of organs and tissues and further recursively modulate the genetic expression. These physical factors are mechanical, chemical, and geometrical, as well as use-related stressors. Here, the distance or contiguity between cells allows for a spatial-dependent gradient of information such that the regulatory signals created by one cell reach neighbouring cells and regulate the surrounding environment depending on the geography of their position. Therefore, deterministic, stochastic, local, and population factors cooperate during self-organisation, which moves towards a reduction of the overall free energy of the system and stabilisation around the steady states.

Our approach, using Kikuchi free energy, endorses this perspective, namely, that regulatory signals created by cells can affect neighbour cells and influence the surrounding cluster. This induces the creation of new homeostasis in the tissue population (or the body) as a new equilibrium is reached. This stipulates that cancer niche construction is not destructive in physical terms but speaks to the natural evolution of the state function of the system. We have observed changes across our three cancer trajectory simulations. Here, cancer niche construction (or a topographic change to the system) persists because it minimises the overall Kikuchi free energy of the system. This has been shown during metastasis and local growth as nodes change from a healthy to a cancerous state here. Similarly, through apoptosis, a new minimum is reached as the system topology shifts from cancerous to an overall healthy state. The new homeostasis is a consequence of the changes in the interaction and tolerance parameters, that influence the overall Kikuchi free energy estimation and regulate the niche construction.

These parameters allow for changes at both the sub-population (or cluster) level and the overall system. Specifically, under this formulation, the overall system state is regulated by the tolerance level that determines the threshold for having cancerous cells. In our simulations, this meant that a system with high tolerance would permit an increased number of cancer nodes that are conducive to the proliferation and growth of cancer niches. Conversely, low tolerance meant that the system could not maintain the existing cancer niche and/or create a new cancer niche. Additionally, the interaction parameter regulates the types of interactions that would minimise the overall Kikuchi free energy. High interaction parameter values allowed for healthy pairwise interactions for cancer in clusters that are favourable for constructing a new niche. A low interaction parameter value shifted the system state towards either healthy or cancerous by preferring either healthy–healthy or cancer–cancer cell interactions. We postulate that these particular parameters can refer to the biochemical elements in the body which induce cancer formation, growth, and metastasis, as well as the activations of mechanisms of defence.

Our work provides cancer system biology research with a quantitative formulation of evaluating cancer niches that speaks to the recent change of perspective in the field. The role of population dynamics and cancer niches has been proven to be at the core of cancer growth. Our model includes these elements and illustrates their importance in evaluating cancer trajectories. Consequently, this approach has promising future directions for the field of computational biology and can help our understanding of how niches and cells and how possible therapies interact and interfere with each other. Similar mathematical approaches could be considered to test and validate the hypothesis, as well as to predict the possible development of a cancer mass depending on the degree of development and growth within its niche. Although our model is just a reductive simplification of the complex process of carcinogenesis, it demonstrates how Kikuchi free energy is a valuable tool for system biology studies.

### 5.1. Other Computational Approaches 

The authors of [42] characterised cancer niche concentration as a (stochastic) transition of a healthy system to a distinct oncogenic steady state, e.g., proliferation or apoptosis. They hypothesised that this transition is a direct consequence of the nonlinear dynamic interactions amongst molecular/cellular pathways and modules, e.g., E2F [43], which constitutes an endogenous network. They introduced nonlinear stochastic differential equations (a generalised form of the Langevin equations) in [44,45,46] to elucidate the specific interactions and nodes that undergird these transitions. See [47] for a review of this approach. This approach is conceptually consistent with a continuous state-space formulation in the current work, albeit introducing stochastic dynamics. Conversely, our approach considers a simplified setting with a discrete-state space formulation with a minimum set of assumptions about the types of nodes and how they interact. Our focus places an endogenous set of agents, as defined by [42], in the setting of cellular population dynamics. This casts the construction of cancer niches in terms of interactions between neighbouring cells and how they influence the cluster using Kikuchi free energy. Interestingly, [47] proposed that free energy can be used to evaluate such noisy systems.

Our approach is complementary to the 2D stochastic cellular automation model proposed in [48] with three states: proliferative, dead (‘vacant’), or quiescent. They proposed distinct (deterministic) transitions between each state with three hyperparameters governing the system dynamics (regrowth ability, death rate, and cell cycle arrest). Using Monte Carlo simulations and mean-field phase transition equations, they suggested that the collapse of homeostasis at the multicellular level may be underwritten by non-equilibrium processes; however, their model did not consider long-range intercellular interactions using Kikuchi free energy. Future work could look to incorporate the dynamic transitions introduced in [48] and update rules to model particular cancer niches using Kikuchi free energy.

### 5.2. Limitations

There are several limitations with our formulation of cancer niche construction as a consequence of the 2D Ising model used to describe our system. The model restricts the simulations to a closed grid that is unable to interact with the “outside” or be affected by external forces [49]. Nonetheless, this is sufficient for the purposes of understanding how the internal dynamics of a tissue population induce cancer niches. Moreover, our work provides a first step in going beyond a (factorised) mean-field approach to evaluating cancer proliferation and growth [7,8,9,10]. A second limitation arises from the deterministic formulation of the transition dynamics. As mentioned above, the self-organisation of cancer niches is a direct consequence of deterministic and stochastic processes that influence the appropriate environment for the growth and maintenance of oncogenic cells. An enactive formulation has been explored in [50] which casts self-organisation as an active inference process where morphogenesis is simply a result of variational free-energy minimization (i.e., of the sort that has been used to explain action and perception in neuroscience [51,52]) and morphogenesis in cellular biology [53].

A key difference between applications of the free energy principle to pattern formation [50] and the treatment in this paper rests upon the nature of the free energy. In applying the free energy principle, the variational free energy pertains to (Bayesian) beliefs parameterised by some (e.g., internal) states about other (e.g., external) states. In contrast, the application of free energy in this paper is directly attributable to the probability of states. This means the free energy principle treats cancer as a process of inference, e.g., a kind of delusion [54], whereas the current treatment treats carcinogenesis as a thermodynamic process (where, under certain conditions, one implies the other); however, future work could look to use Kikuchi free energy under a generalised belief propagation scheme [20] for modelling cancer niches while incorporating (i) external states and (ii) equipping cells with agency by conditioning state transitions on some active states.

Another limitation is a result of the simple generative model, i.e., our discrete random variables can be realised as either cancerous or healthy. Thus, when modelling metastasis, we are unable to model intermediate changes in the cell type (or different realisation of the random variables) as they move from the primary to the metastatic site. Future work could look to expand the model formulation beyond the 2D Ising model to account for these different states as particular cancer niches develop. This would give us a more realistic grounding in the transition dynamics that go beyond being simply healthy to cancerous or cancerous to healthy.

## 6. Conclusions

In this work, we illustrate that cancer niche construction is a direct consequence of interactions between clusters of modified cancerous cells using Kikuchi free energy approximation. We show that for certain cancer trajectories, Kikuchi free energy is a more accurate measure of evaluating system topology when compared to a mean-field approximation. Consequently, our work provides proof for the principle of using higher-order free energy approximations that can be more appropriate when evaluating cancer niche construction. Future work should extend the system formulation beyond a 2D Ising construct to evaluate the underlying differences in free energy approximations.

## Figures and Tables

**Table 1:** Parameterisation of the simulations. The interaction parameter regulates the type of cell interaction. The tolerance parameter determines the acceptance level of cancerous cells. The growth hyperparameter controls the number of cell states allowed to switch during a single trial. The noise hyperparameter influences the transition dynamics. These parameters determine the evolution of the system to a steady state according to Equation (14).

	Tolerance, tol	$\mathrm{Interaction},\;\varepsilon_{IN}$	Growth Rate *, *α*	Noise, *nt*
Local Growth	0.60000	2.77259	0.00610	0.25/0.00
Metastasis	0.60000	1.88001	0.00061% → 0.00610%	0.30 → 0.25
Apoptosis	0.00001	−1.42670	0.00610%	0.25

## Appendix A

### Exact Formulation for the Kikuchi Free Energy

We use the Kikuchi free energy formulation introduced in [16]:

(A1) $$\begin{array}{cc} F_{V} & =U_{K}(Q)-S_{K}(Q)+\lambda_{1}\left(1+\sum_{s_{1},s_{2},s_{3}\in \{0,1\}}\sum_{i,j}H(x_{{}_{i,j}}^{s_{1}},x_{{}_{i-1,j-1}}^{s_{2}},x_{{}_{i-1,j+i}}^{s_{3}})\right)\\ & +4\lambda_{2}\left(\begin{array}{l} \sum_{i,j,j_{1,}{}_{2},i_{i,2}}Q(x_{{}_{i,j}}^{0},x_{{}_{i_{1},j_{1}}}^{1},x_{{}_{i_{2},j_{2}}}^{0})+\sum_{i,j,j_{1,}{}_{2},i_{i,2}}Q(x_{{}_{i,j}}^{1},x_{{}_{i_{1},j_{1}}}^{1},x_{{}_{i_{2},j_{2}}}^{0})\\ -\sum_{i,j,j_{1,}{}_{2},i_{i,2}}Q(x_{{}_{i,j}}^{0},x_{{}_{i_{1},j_{1}}}^{0},x_{{}_{i_{2},j_{2}}}^{1})-\sum_{i,j,j_{1,}{}_{2},i_{i,2}}Q(x_{{}_{i,j}}^{1},x_{{}_{i_{1},j_{1}}}^{0},x_{{}_{i_{2},j_{2}}}^{1}) \end{array}\right) \end{array}$$

where the following may be represented using Equation (1.3) in [16]:

(A2) $$\begin{array}{cc} U(Q) & =\underset{activation}{\underset{︸}{U_{0}}}+\varepsilon_{IN}\left(\begin{array}{l} \sum_{i,j,j_{1,}{}_{2},i_{i,2}}Q(x_{{}_{i,j}}^{0},x_{{}_{i_{1},j_{1}}}^{0},x_{{}_{i_{2},j_{2}}}^{0})+\sum_{i,j,j_{1,}{}_{2},i_{i,2}}Q(x_{{}_{i,j}}^{0},x_{{}_{i_{1},j_{1}}}^{1},x_{{}_{i_{2},j_{2}}}^{0})\\ +\sum_{i,j,j_{1,}{}_{2},i_{i,2}}Q(x_{{}_{i,j}}^{1},x_{{}_{i_{1},j_{1}}}^{0},x_{{}_{i_{2},j_{2}}}^{1})-\sum_{i,j,j_{1,}{}_{2},i_{i,2}}Q(x_{{}_{i,j}}^{1},x_{{}_{i_{1},j_{1}}}^{1},x_{{}_{i_{2},j_{2}}}^{1}) \end{array}\right)\\ & =\varepsilon_{IN}\left(\begin{array}{l} \sum_{i,j,j_{1},i_{1}}Q(x_{{}_{i,j}}^{1},x_{{}_{i_{1},j_{1}}}^{0})+\sum_{i,j,j_{1},i_{1}}Q(x_{{}_{i,j}}^{0},x_{{}_{i_{1},j_{1}}}^{1})-\\ \sum_{i,j,j_{1},i_{1}}Q(x_{{}_{i,j}}^{0},x_{{}_{i_{1},j_{1}}}^{0})-\sum_{i,j,j_{1},i_{1}}Q(x_{{}_{i,j}}^{1},x_{{}_{i_{1},j_{1}}}^{1}) \end{array}\right) \end{array}$$

(A3) $$\begin{array}{cc} S(Q) & =\sum_{s_{1},s_{2},s_{3}\in \{0,1\}}\sum_{i,j}S(x_{{}_{i,j}}^{s_{1}},x_{{}_{i-1,j-1}}^{s_{2}},x_{{}_{i-1,j+i}}^{s_{3}})-\sum_{s_{1},s_{2}\in \{0,1\}}\sum_{i,j}S(x_{i,j}^{s_{1}},x_{i+1,j}^{s_{2}})\\ & -\sum_{s_{1},s_{2}\in \{0,1\}}\sum_{i,j}S(x_{i,j}^{s_{1}},x_{i+1,j+1}^{s_{2}})+\sum_{s_{1}\in \{0,1\}}\sum_{i,j}S(x_{i,j}^{s_{1}}) \end{array}$$

## Appendix B

Pseudocode for simulating our system.

## References

[1] Schofield R. (1978). The relationship between the spleen colony-forming cell and the haemopoietic stem cell. Blood Cells.

[2] Ferraro F., Celso C.L., Scadden D. (2010). Adult stem cels and their niches. Cell Biol. Stem Cells.

[3] Donnelly H., Salmeron-Sanchez M., Dalby M.J. (2018). Designing stem cell niches for differentiation and self-renewal. J. R. Soc. Interface.

[4] Yu Z., Pestell T.G., Lisanti M.P., Pestell R.G. (2012). Cancer stem cells. Int. J. Biochem. Cell Biol..

[5] Plaks V., Kong N., Werb Z. (2015). The cancer stem cell niche: How essential is the niche in regulating stemness of tumor cells?. Cell Stem Cell.

[6] Hanahan D., Weinberg R.A. (2000). The hallmarks of cancer. Cell.

[7] Torquato S. (2011). Toward an Ising model of cancer and beyond. Phys. Biol..

[8] Züleyha A., Ziya M., Selçuk Y., Kemal Ö.M., Mesut T. (2017). Simulation of glioblastoma multiforme (GBM) tumor cells using ising model on the Creutz Cellular Automaton. Phys. A Stat. Mech. Its Appl..

[9] Llanos-Pérez J., Betancourt-Mar J., Cocho G., Mansilla R., Nieto-Villar J.M. (2016). Phase transitions in tumor growth: III vascular and metastasis behavior. Phys. A Stat. Mech. Its Appl..

[10] Barradas-Bautista D., Alvarado-Mentado M., Agostino M., Cocho G. (2018). Cancer growth and metastasis as a metaphor of Go gaming: An Ising model approach. PLoS ONE.

[11] Lei J., Levin S.A., Nie Q. (2014). Mathematical model of adult stem cell regeneration with cross-talk between genetic and epigenetic regulation. Proc. Natl. Acad. Sci. USA.

[12] Ravichandran S., Okawa S., Arbas S.M., Del Sol A. (2016). A systems biology approach to identify niche determinants of cellular phenotypes. Stem Cell Res..

[13] Székely Jr T., Burrage K., Mangel M., Bonsall M.B. (2014). Stochastic dynamics of interacting haematopoietic stem cell niche lineages. PLoS Comput. Biol..

[14] Maren A.J. (2021). The 2-D Cluster Variation Method: Topography Illustrations and Their Enthalpy Parameter Correlations. Entropy.

[15] Yedidia J.S., Freeman W.T., Weiss Y. (2001). Bethe free energy, Kikuchi approximations, and belief propagation algorithms. Adv. Neural Inf. Process. Syst..

[16] Kikuchi R., Brush S.G. (1967). Improvement of the Cluster-Variation Method. J. Chem. Phys..

[17] Kikuchi R. (1951). A theory of cooperative phenomena. Phys. Rev..

[18] Maren A.J. (2016). The cluster variation method: A primer for neuroscientists. Brain Sci..

[19] Yedidia J. (2001). An idiosyncratic journey beyond mean field theory. Advanced Mean Field Methods: Theory and Practice.

[20] Yedidia J.S., Freeman W.T., Weiss Y. (2005). Constructing free-energy approximations and generalized belief propagation algorithms. IEEE Trans. Inf. Theory.

[21] Bach A. (1990). Boltzmann’s Probability Distribution of 1877. Arch. Hist. Exact Sci..

[22] Hinton G.E., Zemel R.S. (1994). Autoencoders, minimum description length, and Helmholtz free energy. Adv. Neural Inf. Process. Syst..

[23] Yoshioka D. (2007). The Partition Function and the Free Energy. Statistical Physics: An Introduction.

[24] Parisi G. (1988). Statistical Field Theory.

[25] Cover T.M. (2012). Elements of Information Theory.

[26] Parr T., Sajid N., Friston K.J. (2020). Modules or Mean-Fields?. Entropy.

[27] Parr T., Markovic D., Kiebel S.J., Friston K.J. (2019). Neuronal message passing using Mean-field, Bethe, and Marginal approximations. Sci. Rep..

[28] Kadanoff L.P. (2009). More is the same; phase transitions and mean field theories. J. Stat. Phys..

[29] Jordan M.I., Ghahramani Z., Jaakkola T.S., Saul L.K. (1998). An introduction to variational methods for graphical models. Learning in Graphical Models.

[30] Frey B.J., MacKay D.J. (1998). A revolution: Belief propagation in graphs with cycles. Advances in Neural Information Processing Systems.

[31] Pelizzola A. (2005). Cluster variation method in statistical physics and probabilistic graphical models. J. Phys. A Math. Gen..

[32] Yedidia J.S., Freeman W.T., Weiss Y. (2003). Understanding belief propagation and its generalizations. Explor. Artif. Intell. New Millenn..

[33] MacKay D.J. (2001). A conversation about the Bethe free energy and sum-product. Tech. Rep. of Mitsubishi Electric Research Lab..

[34] Geiger T.R., Peeper D.S. (2009). Metastasis mechanisms. Biochim. Biophys. Acta (BBA) Rev. Cancer.

[35] Elmore S. (2007). Apoptosis: A review of programmed cell death. Toxicol. Pathol..

[36] Homma N., Happel M.F.K., Nodal F.R., Ohl F.W., King A.J., Bajo V.M. (2017). A Role for Auditory Corticothalamic Feedback in the Perception of Complex Sounds. J. Neurosci..

[37] Manicka S., Levin M. (2019). Modeling somatic computation with non-neural bioelectric networks. Sci. Rep..

[38] Cervera J., Manzanares J.A., Mafe S., Levin M. (2019). Synchronization of Bioelectric Oscillations in Networks of Nonexcitable Cells: From Single-Cell to Multicellular States. J. Phys. Chem. B.

[39] Levin M. (2014). Endogenous bioelectrical networks store non-genetic patterning information during development and regeneration. J. Physiol..

[40] Levin M. (2013). Reprogramming cells and tissue patterning via bioelectrical pathways: Molecular mechanisms and biomedical opportunities. Wiley Interdiscip. Rev. Syst. Biol. Med..

[41] Collinet C., Lecuit T. (2021). Programmed and self-organized flow of information during morphogenesis. Nat. Rev. Mol. Cell Biol..

[42] Ao P., Galas D., Hood L., Zhu X. (2008). Cancer as robust intrinsic state of endogenous molecular-cellular network shaped by evolution. Med. Hypotheses.

[43] Weinberg R.A. (1995). The retinoblastoma protein and cell cycle control. Cell.

[44] Yuan R., Zhu X., Radich J.P., Ao P. (2016). From molecular interaction to acute promyelocytic leukemia: Calculating leukemogenesis and remission from endogenous molecular-cellular network. Sci. Rep..

[45] Li S., Zhu X., Liu B., Wang G., Ao P. (2015). Endogenous molecular network reveals two mechanisms of heterogeneity within gastric cancer. Oncotarget.

[46] Zhu X., Yuan R., Hood L., Ao P. (2015). Endogenous molecular-cellular hierarchical modeling of prostate carcinogenesis uncovers robust structure. Progress Biophys. Mol. Biol..

[47] Yuan R., Zhu X., Wang G., Li S., Ao P. (2017). Cancer as robust intrinsic state shaped by evolution: A key issues review. Rep. Prog. Phys. Phys. Soc. (Great Britain).

[48] Lou Y., Chen A., Yoshida E., Chen Y. (2019). Homeostasis and systematic ageing as non-equilibrium phase transitions in computational multicellular organizations. R. Soc. Open Sci..

[49] Friston K.J., Daunizeau J., Kilner J., Kiebel S.J. (2010). Action and behavior: A free-energy formulation. Biol. Cybern..

[50] Friston K., Levin M., Sengupta B., Pezzulo G. (2015). Knowing one’s place: A free-energy approach to pattern regulation. J. R. Soc. Interface.

[51] Friston K., FitzGerald T., Rigoli F., Schwartenbeck P., Pezzulo G. (2017). Active Inference: A Process Theory. Neural Comput..

[52] Pezzulo G., Rigoli F., Friston K.J. (2015). Active Inference, homeostatic regulation and adaptive behavioural control. Prog. Neurobiol..

[53] Kuchling F., Friston K., Georgiev G., Levin M. (2019). Morphogenesis as Bayesian inference: A variational approach to pattern formation and control in complex biological systems. Phys. Life Rev..

[54] Levin M. (2019). The Computational Boundary of a “Self”: Developmental Bioelectricity Drives Multicellularity and Scale-Free Cognition. Front. Psychol..

